# Human resource crises in German hospitals—an explorative study

**DOI:** 10.1186/s12960-015-0032-4

**Published:** 2015-05-28

**Authors:** Carsten C Schermuly, Michael Draheim, Ronald Glasberg, Vladimir Stantchev, Gerrit Tamm, Michael Hartmann, Franz Hessel

**Affiliations:** SRH Hochschule Berlin, Ernst Reuter Platz 10, 10587 Berlin, Germany

## Abstract

**Background:**

The complexity of providing medical care in a high-tech environment with a highly specialized, limited labour force makes hospitals more crisis-prone than other industries. An effective defence against crises is only possible if the organizational resilience and the capacity to handle crises become part of the hospitals’ organizational culture. To become more resilient to crises, a raised awareness—especially in the area of human resource (HR)—is necessary. The aim of this paper is to contribute to the process robustness against crises through the identification and evaluation of relevant HR crises and their causations in hospitals.

**Methods:**

Qualitative and quantitative methods were combined to identify and evaluate crises in hospitals in the HR sector. A structured workshop with experts was conducted to identify HR crises and their descriptions, as well as causes and consequences for patients and hospitals. To evaluate the findings, an online survey was carried out to rate the occurrence (past, future) and dangerousness of each crisis.

**Results:**

Six HR crises were identified in this study: staff shortages, acute loss of personnel following a pandemic, damage to reputation, insufficient communication during restructuring, bullying, and misuse of drugs. The highest occurrence probability in the future was seen in staff shortages, followed by acute loss of personnel following a pandemic. Staff shortages, damage to reputation, and acute loss of personnel following a pandemic were seen as the most dangerous crises.

**Conclusions:**

The study concludes that coping with HR crises in hospitals is existential for hospitals and requires increased awareness. The six HR crises identified occurred regularly in German hospitals in the past, and their occurrence probability for the future was rated as high.

## Background

In industrialized countries, hospitals are the backbone of the health-care system. In Germany, 18 787 168 hospital treatments were conducted in 1 996 hospitals in 2013 [1]. Like in most countries, nearly half of the hospital beds are in public ownership with a growing number of privately owned hospitals [2]. The aim of the hospitals is to heal diseases, prevent deterioration of the patients, or alleviate disease symptoms through trained staff and adequate equipment. For that reason, hospitals are a relatively hazardous working environment for staff. But the complexity of providing medical care in a high-tech environment makes hospitals more crisis-prone than less complex industries [3]. Due to the rising incidence and impact of crises, the significance of crisis research has heightened in recent years [4]. However, there is still a lack of generally agreed definitions and typologies of crises [4, 5]. A crisis is “a state differing from the normal state which has the potential to or already has incurred damage to persons and/or technical equipment” [6]. Transferred to hospital management, a crisis is one or numerous critical situations which could not be handled by routine measures of quality management. A hospital crisis is regarded as an event or a series of events that may occur either suddenly or which may take some time to evolve. It results in a major, urgent problem with potentially severe consequences for the hospital, and it must be addressed immediately.

Although crisis management activities have increased significantly in recent years, systematic reviews indicate that the scientific evidence for safety interventions in hospitals is still limited and that the methodological quality of the studies is generally weak [3, 5, 7–10]. A US nationwide survey that was using a sample of 939 hospitals illustrated that there are some opportunities for improvement in hospital crisis management [5]. The researchers demonstrated that hospitals in rural areas are less prepared for crises than hospitals in urban areas. Moreover, they noted that current standards and crises plans are papers that are commonly placed in the shelf when a crisis occurs and the implemented standards provide no guarantee or assurance that the crisis response will be adequate. In addition, they showed that no outcome measures exist to determine if a crisis plan will be followed or is effective. These findings are supported by the research of Rebmann et al. [9] who identified that US hospital staff is often not updated on crisis plan changes.

Due to the high-personnel density in hospitals, crises related to human resource (HR) issues are very important for the safety of patients and the success of hospitals. Although there is a growing number of publications addressing crises und crisis-preparedness in hospitals [3–5, 7–9, 11–13], literature related to HR crises in hospitals is scarce: only a few publications deal with the topic of crisis impact on HR management in hospitals [10, 14]. A systematic review identified gaps in staff education for adequate crisis response and discovered that hospital crisis prevention plans are not well-implemented or standardized [15]. Another review underlines these findings, as well as the insufficient evidence for the effectiveness of methods to train staff in crisis response [14]. Also, the results from a survey of 663 hospital managers show that there is a lack of the provision of staff when a crisis occurs [9].

In comparison to other industries, hospitals must still improve on identification, analysis, evaluation, and control of crises within hospital facilities [16, 17]. The literature research shows that an effective defence against crises is only possible if the capacity to handle them becomes a more important part of the hospitals’ organizational culture [18, 19]. This is why the transition to a crisis resolution culture is recommended. To become more resilient to crises, a raised awareness of crises, especially in the area of HR, is necessary [19].

With the present research, we intend to address several research gaps and develop useful knowledge for practitioners. First, we want to identify specific crisis scenarios that are perceived as most relevant by hospital care providers, because no publications exist that provide hospital executives with an overview of HR crises, which may occur in their hospitals. Second, we want to identify causes for these HR crises as knowledge about the causes of crises can help to develop prevention strategies. Third, we want to empirically quantify the occurrence of these crises in the past and try to give information about the likelihood of occurrence in the future, as well as about their perceived dangerousness. This knowledge supports the selection of those crises, which are most important and should consequentially receive special attention in practice and research.

Because of the limited state of research, we chose an explorative research strategy that combines qualitative and quantitative methods. The crises were identified in workshops with experts in the field while their causes distinguished through semi-structured interviews. The past and future occurrences as well as the dangerousness were empirically investigated in a sample of 74 hospitals in Germany.

## Methods

Qualitative and quantitative methods were combined to identify and evaluate crises in hospitals in the HR sector. Qualitative approaches were used in the first step to identify relevant HR crises and their causes as well as to compile a description of these crises. The qualitative approach was used because we could not revert to studies that had already investigated this in detail in the past and because we needed detailed information about the nature of each crisis. Thereafter, we worked quantitatively and evaluated these crises empirically to gain general knowledge about the occurrence and the dangerousness of these crises in the German hospital market.

In order to draw reliable and valid knowledge from German hospitals, relevant experts had to be found. To identify experts for our workshop and the quantitative survey, addresses were obtained from the hospital register which is administered by the German Federal Office of Statistics. For every hospital (>50 beds), a contact person (executive level) was identified through a phone call or internet research. This person was asked to participate at our expert workshops and/or answer the questionnaire or appoint the responsible expert in the hospital. Only one link to the survey was sent to every hospital to ensure that only one person per hospital answers.

Twelve experts participated in the workshop to identify relevant crises while two experts participated in the structured interviews to collect causes for the occurrence of the crises (qualitative pre-studies). Seventy-four experts from 74 hospitals evaluated the past and future occurrence of the crisis through an online survey. We did not conduct any experiments. Our research was also carried out on organizations (hospitals) and not humans. All ethical guidelines were respected.

### Qualitative pre-studies to identify crises and their causes

As a result of the limited current state of the research, structured workshops with experts from the health-care sector were conducted in order to identify HR crises in hospitals. Twelve experts from German hospitals participated in the workshop. Three were HR directors, two were heads of an organizational department, two professors, one senior physician, one executive director, and one assistant to a hospital executive director. One participant directed the security management and another participant oversaw the quality management department in their respective hospital.

The workshop was moderated by a university professor of business psychology with expertise in workshop and moderation methods. He was supported by a co-trainer and one other professor. The workshop lasted for 5 h. First, the participants were introduced to the topic by the moderator. The project, the course of the workshop, and the definition of crises in hospitals (“a state differing from the normal state which has the potential to or already has incurred damage to persons and/or technical equipment”) were presented [6]. Afterwards, the participants worked individually for 10 min and were asked to write down at least five HR crises on a prepared handout. During a break, all crises were written on cards, clustered, and transferred onto a pin board. Following the break, the crises were presented, and the participants received 10 red points to evaluate the crises. The participants had to assign these to a certain crisis, depending on how negatively they interpreted the impact of a particular crisis on a hospital and for which crisis they wished to be prepared for. Afterwards, the six crises, which had received the highest number of points, were selected. Experts were paired and given 1 h to work together. Each team was working on one crisis with the objective to develop core characteristics as well as the main consequences and costs of the crisis. In the last part of the workshop, the results for each crisis were presented, and the other experts had the opportunity to add important aspects to each crisis and discuss the findings. The summarized results are presented in Table 1.

**Table 1:** Identified crises and their main characteristics

Crisis	Main characteristics
Staff shortages	• Limitation of certain skills within the HR market
	• Applicants do not meet requirements for advertised positions
	• Vacant positions cannot be filled
	• Lack of staff restricts operations
	• Service plans cannot be ensured, and patients must be sent away
Acute loss of personnel following a pandemic	• A pandemic refers to a quickly spreading infection spanning a large area
	• Hospitals are particularly affected by pandemics because:
	➢ Larger workload due to increasing number of patients
	➢ Employees are exposed to and affected by the pandemic reducing the number of effective staff
	• Spontaneous pandemic can result in a lack of personnel
Damage to reputation, internal information is made public	• Distribution of internal information regarding the hospital
	• Rumours are inflated and misrepresented
	• Results in unrest among employees
	• Rumours impart a negative light on hospital operations and future recruiting of skilled employees
Insufficient communication during restructuring	• Within restructuring measures, it is understood that organizational and financial measures will be required to reinstate the performance capacities of insolvent companies
	• Measures are often not sufficiently communicated to those both directly and indirectly affected
	• Those affected are not included in communications regarding restructuring
	• Resentment among employees can lead to decreased motivation
Bullying	• Bullying causes conflict-ridden communication within a team where a victim is systematically directly/indirectly attacked from antagonists over a period of time
	• A senior staff member is accused to be a suspect
	• Accusations result in a burden for other hospital staff
	• Can lead to a rise in staff sick leave
Misuse of drugs by a superior	• There is misuse of medication in the hospital setting
	• These may also be misuse or use of alcohol or other drugs
	• An employee in leadership position or senior physician is affected
	• The first signs of an addiction are noticed
	• This leads to mistrust and decreased respect for the victim

In a second step, the causes for each crisis were ascertained.

The identification of the causes was conducted in two steps: in the first step, one semi-structured telephone interview with a director of the security management of a hospital was organized and conducted; the other one was held with an expert in personnel controlling. The interviews started with the presentation of the workshop results (see Table 1). After that, the participants were asked which causes could lead to that particular kind of crisis in a hospital. Concurrently, a literature search to identify causes from relevant publications was conducted in the following databases: PubMed, EconBiz, and PsycINFO. The collected information was processed (discussed and evaluated in the research team) and included in the survey.

### Quantitative survey

#### Sample

Seventy-four hospitals participated in the online survey. Among the participating hospitals, the median of full-time employees is 410 (first quartile: 147; third quartile: 865), and the median of patient beds is 275 (first quartile: 134; third quartile: 500). The hospitals were located in 13 of the 16 federal states of Germany (Saarland, Brandenburg, and Schleswig-Holstein are missing) and 35.1% were in public ownership. In 40.5% of the cases, institutions were independent non-profit organizations (e.g. a church-owned hospital), and 24.3% were privately owned.

Employees responding to the online survey were 47.26 (SD = 8.78) years old on average; 20.3% were directors of a hospital, 16.2% were heads of administration, 16.2% were department managers, 10.8% worked as medical staff, 5.4% as care directors, and 31.1% answered other. The respondents who indicated “other” had to fill in their job description. Among them were chief medical officers, top doctors, commercial directors, and quality management officers, as well as crisis management officers.

#### Procedure

The online survey was conducted between October and December 2014. First, an introduction text was presented to the participants. They were welcomed, and the survey procedure was presented. Subsequently, demographic information regarding the hospitals and participants was requested. Afterwards, each crisis was introduced with the text presented in Table 1. After having presented a crisis, the participants had to evaluate the following questions for each crisis:

- *Occurrence* (*past*): “Did this crisis occur in your hospital in the last five years?”
- *Occurrence future* (*own hospital*): “How high is the probability that this crisis will occur in your hospital within the next five years?”
- *Occurrence future* (*other hospitals*): “Think about another optional hospital in Germany: How high is the probability that this crisis will occur in this hospital within the next five years?”
- *Dangerousness*: “How dangerous is this crisis for your hospital?”

The first item had to be answered on a five-point Likert scale (1 = complete, 2 = nearly complete, 3 = partially, 4 = in few divisions, 5 = no). For the second and third item, the participants had to move a slider from 0% to 100%. The last item had to be answered on a seven-point Likert scale from absolutely not dangerous (1) to extremely dangerous (7). Afterwards, the acquired causes of each crisis were introduced with the following sentence: “How important do you evaluate this event for the occurrence of the crises?” Answers were made using a seven-point Likert scale from 1 = absolutely unimportant to 7 = extremely important.

## Results

In the following, the results for the three aims of the study (identification of HR crises and determination of their causes as well as the quantification of their occurrence) are presented. The 12 experts identified 6 relevant crises in the workshop: Staff shortages, acute loss of personnel following a pandemic, damage to reputation, internal information is made public, insufficient communication during restructuring, bullying, and misuse of drugs by a superior. The main characteristics prepared in the workshops are presented in a table. For example, an acute loss of personnel following a pandemic possesses the characteristic that not only more patients have to be treated but also that less staff is available because more employees are infected, too.

The results of the survey in which 74 hospitals participated are displayed in Table 2.

**Table 2:** Occurrence (past and future) and dangerousness of each crisis

Crisis	Risk indicator^a^	Occurrence (past) 1 complete to 5 no (mean)	Occurrence (future) in percent (mean)		Dangerousness (mean)
			Own	Other	
Staff shortages	220.09	3.99	46.1	57.9	4.8
Acute loss of personnel following a pandemic	176.67	4.33	37.4	43.4	4.7
Damage to reputation, internal information is made public	168.55	4.16	35.6	46.9	4.7
Insufficient communication during restructuring	140.72	4.05	34.5	54.9	4.1
Bullying	132.97	4.15	35.3	47.5	3.8
Misuse of drugs by a superior	115.75	4.34	30.3	48.2	3.8
Mean			36.5	49.8	4.3

^a^Occurrence (future) own hospital multiplied with dangerousness

**Fig. 1:**
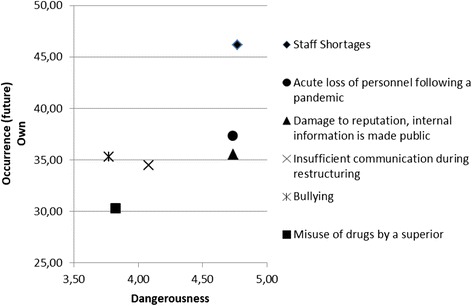
Occurrence (future) own hospital and dangerousness of each crisis

To rank the different crises, an indicator was calculated. The indicator derives from the definition of “risk” in literature, where the measurement of risks consists of two components. These two components are the probability of occurrence and the threat intensity. This measure of the risk of an event is broadly used in economic and health economic literature and provides an indicator to rank the different scenarios [6, 20–26]. We operationalized the probability of occurrence with item 2 (evaluation of future occurrence in one’s own hospital) and threat intensity with item 4 (evaluation of dangerousness). Both values were multiplied to receive an indicator for the severity of a crisis. This multiplication shows the following results (see Fig. 1): staff shortages (220.09), acute loss of personnel following a pandemic (176.67), damage to reputation (168.55), insufficient communication during restructuring (140.72), bullying (132.97), and misuse of drugs (115.75). Because this risk indicator is the most important value to evaluate a crisis for us, we analysed if there are mean differences between the six crises. Each participant evaluated each crisis in a serial order, and so, the evaluations are not independent. That is why a repeated measure ANOVA was calculated. Mauchly’s sphericity test revealed significant variance differences. Thus, we relied our significance testing on the Greenhouse–Geisser procedure, which showed significant mean differences between the risk indicators (*F* (4.2) = 7.42; *p* <0.001). Afterwards, we conducted *post hoc* tests and used a Bonferroni correction which is a conservative procedure to handle the problem of alpha inflation. There were, for example, no significant mean differences between the crisis of staff shortages and the second highest risk indicator (acute loss of personnel following a pandemic; *p* >0.05) as well as the third highest (damage to reputation; *p* >0.05), but there were significant mean differences between staff shortages and all other crises (*p* <0.01).

In the second column, the occurrence over the past (last 5 years) is shown. The crises occurring most frequently are staff shortages and insufficient communication during restructuring, followed by bullying, reputation damage, the acute loss of personnel following a pandemic, and misuse of drugs by a superior.

In the next column, the future occurrences of the crises for one’s own hospital are shown. The highest probability was seen for staff shortages (46.1%) followed by acute loss of personnel following a pandemic (37.4%). The lowest probability was seen for drug misuse by a superior (30.3%). The future occurrence probabilities for other hospitals are ranked higher than for one’s own hospital (*M* = 36.5% versus *M* = 49.8%). Staff shortages, damage to reputation, and acute loss of personnel following a pandemic were seen as the most dangerous crises (see last column in Table 2). Bullying was seen as the crisis with the lowest dangerousness.

The results for the causes identified in the semi-structured interviews and the evaluation in the survey are displayed in Table 3. The causes for each crisis are structured according to their rated importance for the occurrence of the crisis. Our results indicate that demographic change and sparsely populated areas are seen as the most important causes for staff shortages. Loss of personnel because of a pandemic can be explained by a lack of staff, high-work strain, and poor hygiene. Dissatisfied employees and a lack of protective measures have the highest importance rate for hospital reputation during a crisis. Participants identified under-appreciation of effective communication within the hospital and addressing the wrong target group as important causes for communication problems during crises. Lack of management and conflicts within the team were identified as the most important causes for bullying, and easy access to medication and strenuous workload were identified as the most important causes leading to misuse of drugs. All other evaluations are shown in Table 3.

**Table 3:** Evaluated causes of crises

Crisis	Cause	*M*	SD
Staff shortages	Demographic change	5.36	1.55
	Sparsely populated area	5.10	1.70
	Insufficient immigration of skilled personnel	4.66	1.68
	Poor working conditions	4.58	1.97
	High incidence of occupational illness	3.13	1.54
Acute loss of personnel following a pandemic	Lack of personnel	4.53	2.06
	High-work strain (e.g. forced to work despite being ill).	4.24	1.99
	Poor hygiene	4.16	1.89
	Other plausible attack on staff health	3.05	1.88
Damage to reputation, internal information is made public	Dissatisfied employees	5.75	1.54
	Lack of data protection measures	4.10	1.94
	Sabotage	3.93	2.09
	Business interests of competitors	2.80	1.61
Insufficient communication during restructuring	Under-appreciation of effective communication	5.85	1.19
	Inappropriate target group	5.52	1.28
	Poor communication timing	5.21	1.48
	Lack of training in effective communication	5.19	1.46
	Conflict among staff	4.93	1.47
Bullying	Poor or lack of management	5.90	1.36
	Conflict within the team	5.74	1.37
	Jealousy, frustration	5.54	1.47
	Unclear tasks and responsibilities	4.94	1.66
	High workload	4.78	1.86
Misuse of drugs by superior	Easy access to medication	5.36	1.78
	Strenuous workload	4.73	1.94
	Protection of a colleague	4.53	1.82
	Increased liberalization (attitudes, approaches, etc.)	3.41	1.77

*M* mean; *SD* standard deviation

## Discussion

This study had three aims: firstly and secondly, HR crises in German hospitals and their causes were identified in an explorative way. Thirdly, information about the occurrence (past and future) and the dangerousness of these relevant crises were collected empirically. To accomplish these goals, several methodical steps were conducted. The crises were ascertained in workshops (12 participants), and the causes were identified in semi-structured interviews with experts from the field (2 participants). The empirical evaluations of the crises were conducted nationwide via an online questionnaire (74 participants). In this discussion, firstly, the qualitative and then the quantitative results will be discussed. The results regarding the causes are used to derive practical implications. Finally, the limitations and the conclusion are presented.

Six different HR crises were seen as relevant for German hospitals from our experts’ point of view (see Table 1). According to the experts, staff shortages are characterized by the fact that certain skills are not available within the HR market. Positions are vacant, but the applicants do not have the requested competencies. As a consequence, vacancies cannot be filled, service plans cannot be ensured, and patients must be sent away. This description is close to other definitions of staff shortages. For example, Buchan and Aiken [27] describe staff shortage as a gap between the reality of current availability of staff and the aspiration for some higher level of provision. The fact that staff shortages are seen as a “crisis” raises the question about the nature of a crisis. Staff shortage is surely an abnormal situation which can threaten the operation, safety, and reputation of a hospital (see definition above), but the sudden and unforeseeable character, which is typical for many crises, is not the case here.

This is more appropriate for the second crisis. As the experts determined in the workshop, a pandemic is a special crisis for hospitals because the workload is higher than during regular operations, and at the same time, the number of employees decreases. For example, nearly half of the 2002 SARS epidemic cases in Toronto and Singapore were detected in health-care workers [11]. A pandemic, however, is only one reason for the occurrence of increased numbers of patients and affected hospital staff at the same time. Mass casualty events or natural disasters like a hurricane or an earthquake can cause a similar crisis [9, 28].

In the third crisis, the experts described that it is a relevant crisis when important internal information from the hospital are made public. According to our experts, the consequences cause unrest among employees and shed a negative light on the hospital, which can result in problems to recruit skilled employees in the future. Here, a connection can be seen between the first and the third crisis. But negative information can also influence patients to choose another hospital resulting in an outflow of patients.

“One of the features that characterize contemporary organizations is change” ([29], p. 46). This is shown in the fourth crisis in Table 1. There are many different occasions for organizational change in hospitals and the restructuring of the organizational system (e.g. fusion of two hospitals, expansion of offered services, new IT systems, or a changed market situation). Our experts identified one key problem, which can endanger these organizational changes: the insufficient communication of measures to those affected by the organizational change.

Bullying as the fifth crisis in Table 1 is typically defined as “hostile and unethical communication, which is directed in a systematic way by one or a few individuals mainly towards one individual…” ([30], p. 168). This is close to the description of the experts in Table 1. Additionally, they emphasize the consequences of bullying (burden for other staff and increased rates of sick staff) and the central position of supervisors. This is in accordance with empirical research. In a large German sample, it was found that 50% of the delinquents were supervisors who acted alone or together with a group of employees [31].

Finally, the misuse of drugs by superiors such as senior physicians was identified as an important crisis. The experts emphasized that not only medications but also alcohol and other drugs can be involved. [32]. It is estimated that 10%–14% of US physicians may become dependent at some point in their career (when alcohol is excluded, the rate is reduced by 1% to 2% [33]). Especially anesthesiologists are overrepresented regarding chemical drugs because of greater access in the workplace [33].

The empirical quantification of the crises was another aim of this study. That is why the empirical results are going to be discussed in the next step. The highest risk indicator value was found for staff shortages (see Table 2). The prominence of this topic might be due to the special situation in Germany. For many years, Germany has one of the lowest birth rates in Europe. Calculations from the WHO indicate that there will be a lack of 12.9 million health-care workers by 2035 worldwide [34]. In the German health-care system, there is a predicted deficit of about 950 000 health-care workers [35]. An investigation in German hospitals revealed that the proportion between vacant and occupied positions will increase from 2% in 2011 to 15% in 2030 [35].

The high position of a pandemic might be influenced by the recent prominence of this topic in the media. The data collection was conducted before the recent outbreak of the Ebola virus, but also, other pandemics like SARS were strongly in the focus of the media. Additionally, a preparation for a potential pandemic outbreak is not only a relevant topic for hospitals but also for the national authorities [36, 37]. Thus, there is already a growing number of studies addressing topics related to capacity planning [38] and preparedness for bioterrorism and other infectious diseases [9, 11–13, 39].

As explained above, the risk indicator is the product of the occurrence probability of the crisis in the future, multiplied with its dangerousness. The occurrence in the past is no part of this value. As shown in Table 2, there is another order for the occurrence in the past. Insufficient communication during restructuring attains the second position. This might also be due to a special situation in Germany. The hospital market in Germany changed a lot over the last decades: Since 1993, the number of hospitals in Germany has been decreasing continuously. At the same time, many hospitals changed from public into private sponsorship. The proportion of beds in private hospitals increased about 66% from 2003 to 2013 [40]. At the same time, the number of beds in public hospitals decreased about 17%. Schmid and Ulrich found that a large number of German hospital markets are characterized by high degrees of market concentration [41]. This trend could have led to many organizational changes and maybe a lot of crises, which were perceived from our participants regarding the past of their hospitals.

In general, the results illustrate that the representatives of the hospitals expect the identified crises to occur quite often in the next 5 years. The average occurrence probability is estimated to be 36.5% on average with a rather high dangerousness (*M* = 4.3). In other hospitals, the occurrence probability within the next 5 years is even perceived as nearly 50% on average. The gap between one’s own and other hospitals might be explained by a psychological attribution effect called self-serving bias (the tendency to see oneself better than others [42]). Nevertheless, if the estimated percentage for own hospital crises is correct, it is still very high. This is why practical implications should be made in the next part of the discussion section.

### Practical implications

For the practical implication, the results for the causes (Table 3) and general research knowledge for each crisis should be used. We start with the crisis with the highest risk indicator: staff shortages. Reasons for staff shortage result from a demographic imbalance that leads to an increased demand in health-care supply on the one hand and from an ageing health-care workforce on the other [43]. Furthermore, there is a declining share of younger people facing an increasingly older population [27, 44]. Aside from the demographic imbalance, which was identified as the main reason for the crisis, there is a decline of health-care staff, especially of nurses [45]. In a large European study, which examined nurses’ early occupation exits with a sample of 39 898 European nurses, reasons for the lack of staff were identified. Many nurses stated physical and mental stress and lack of staff and time to provide adequate care to patients as reasons to leave the job early [45]. In addition, early retirement, career changes to better paid jobs, or emigration also lead to increasing shortage of staff [46].

Measures to attenuate the effects of staff shortage in a hospital must address the causes of the crisis. Poor working conditions can be mitigated with more attractive opportunities to develop on a professional level, to gain autonomy, and to participate in decision-making while being fairly rewarded [34]. Furthermore, there is a need to increase the political and technical leadership for policymakers to support long-term human resource development efforts. One identified cause for staff shortage—low immigration of skilled personnel—could be solved by increasing the number of apprenticeships and university placements or promoting the immigration of skilled workers [34, 47]. Addressing demographic change as the main cause for staff shortage is a long-term social challenge that requires political support. Additionally, mechanisms for the voices, rights, and responsibilities of health workers in the development and implementation of policies and strategies towards universal health coverage should be provided [34].

The lack of staff was identified as one of the main causes for staff shortage during a pandemic. A central inventory of all clinical staff with their current roles and provision of re-trainings can be one opportunity to face the increased demand of medical staff in hospitals during a pandemic. The roles and responsibilities of key individuals during pandemics or disasters should be clearly defined [48]. Furthermore, interim staffing can be one solution to face a short-time increased demand [49]. In order to tackle high workload, which is another identified cause for the acute loss of personnel during a pandemic, HR management should focus on staffing needs like housing, family support, psychological support, and child care when regular child care facilities are closed simultaneously during such scenarios. [9, 48]. Last, but not least, education and trainings for such situations should be enforced.

If damage is done to a hospital’s reputation because internal information was made public, a phenomenon called counterproductive work behaviour (CWB) has occurred. CWB has recently received much attention from researchers and organizations [50]. Due to CWB, 45% of the companies in the US have been subject to one or more significant economic crimes like false pretences or asset misappropriation. Studies showed that personality traits and situational variables affect CWB [50–52]. To address dissatisfied employees as the main cause for this identified crisis, maintaining communication and feedback, allowing participation of employees, and supervisory training are recommended factors to mitigate CWBs [53, 54].

Employee resistance and the associated cognitive and affective processes are seen as the most common problems during organizational change [55]. One opportunity to prevent resistance in organizational change is participation [56]. A component of participation is adequate communication [57]. Neglecting effective communication, inappropriate target groups, and poor communication timing are seen as important causes. The right people (i.e. supervisors or committee members as important multipliers) should be informed at the right time with the right information (e.g. purpose, content, and consequences of change) and via the right communication channels (e.g. personal calls or change workshops). To guarantee this, change agents require complex communication competencies.

In a large Belgian sample (*n* = 6 175), the antecedents of bullying were analysed: for example, role conflicts and role ambiguity as well as high workload, changes in the job, job insecurity, lack of involvement in decision-making, and a lack of skill utilization could be identified as antecedents of bullying at work [58]. These general antecedents are quite in line with the reasons identified by our experts (see Table 3). Hospitals should therefore ensure a highly qualitative and active management and prevent frustration among their staff. Supervisors and team members should be trained in conflict management skills while tasks and responsibilities should be communicated clearly in order to prevent role conflicts and ambiguities.

Drug abuse of superiors such as senior physicians is dangerous. They have direct contact with patients, and the drug abuse can influence treatment and diagnostic quality. Furthermore, senior physicians are in a powerful position and act as role models. Our results indicate that actions to decrease workload (e.g. fewer night shifts) or the experienced workload (e.g. stress management programmes) might be effective measures. Easy access should also be limited, and the organizational culture should aim to support colleagues and supervisors with drug problems.

### Limitations

The current study has some limitations. Even though parallels can be seen with other countries, the results are, strictly spoken, only valid for the German hospital market. Further, the crises as the basis material of this study were identified in a qualitative explorative way with a limited number of participants. This might influence the generalizability of the results. Additionally, the number of participating hospitals is not high enough for a representative sample. Another limitation in our methodical design is that not only the selection but also the evaluation of the crises can be affected by actual news coverage and current problems in the world. Finally, the definition of the item “Dangerousness” can be discussed. This could be interpreted in different directions from different respondents. On the one hand, it can have consequences on monetary aspects; on the other hand, it can have a focus on physical injuries. We decided to use the categorization and the term “dangerousness” which was approved by the research team. The evaluation of costs of HR crises in hospitals could be a point for further research activities. Future research should also pursue a more deductive research strategy and postulates as well as test hypotheses: which crises will occur, when, how, and with which consequences.

## Conclusions

Adequate coping capacities for HR crises in hospitals are important for the success of hospitals and the well-being of staff and patients. In order to deal with crises, crisis resolution capacity is recommended. An important step to crisis resolution capacity is raised awareness for crises. Knowledge about the various types of crises is, therefore, important. Six HR crises were identified in this study: staff shortages, acute loss of personnel following a pandemic, damage to reputation, insufficient communication during restructuring, bullying, and misuse of drugs by a superior. These crises occurred relatively frequently in German hospitals in the past, and their occurrence probability for the future was estimated to be quite high; especially in other hospitals when compared to one’s own. At the same time, however, a manifold number of causes for crises could be identified in this study. This knowledge is useful to prevent future crises in the HR sector.
